# Epidemiological, Clinical and Viral Genomic Features of Influenza Virus Infections Among Pilgrims During the Grand Magal of Touba Over 7 Years (2018–2024)

**DOI:** 10.1002/jmv.71120

**Published:** 2026-08-24

**Authors:** Coumba Diouf, Safietou Diallo, Ihssane Ouaddane, Dame Ndiaye, Ndiaw Goumballa, Déguéne Fam, Mamadou Beye, Clotilde Mauguin, Vincent Bossi, Julien Andreani, Hubert Bassene, Philippe Colson, Philippe Gautret, Cheikh Sokhna

**Affiliations:** ^1^ Aix Marseille Univ, IHU Méditerranée Infection, RITMES Marseille France; ^2^ IRD, UCAD, MINES Dakar Sénégal; ^3^ Aix Marseille Univ, IHU Méditerranée Infection, MEPHI Marseille France

**Keywords:** Africa, genome, Grand Magal of Touba, influenza virus, phylogenomics, Senegal

## Abstract

Large international gatherings such as the Grand Magal of Touba in Senegal may favor influenza A/B virus (IABV) transmission. We analyzed IABV genomes from human samples collected during the event in 2018–2024. Next‐generation sequencing was performed for 190 qPCR‐positive nasopharyngeal samples. Bioinformatic analyzes were performed using MAFFT v7.505, IQ‐TREE v2.2.0, iTOL, Nextclade, and PREDAV‐FluA. 106 (55.8%) complete genomes were obtained, including 99 type A and 7 type B. IAV genomes were classified as A/H3N2 (*N* = 48) and A/H1N1 (*N* = 43), based on hemagglutinin/neuraminidase‐encoding segments. A/H3N2 was not detected in 2021 and 2023; A/H1N1 was only detected in 2018, 2021 and 2023. Phylogenies classified A/H3N2 and A/H1N1 into distinct clades varying from 1 year to the next. A single A/H1N1 sequences was predicted to be antigenic divergence from the vaccine strain concomitantly used. All IBV genomes (clades V1A.3a or V1A.3a.2) were from 2020. Some IABV genomes from the GMT were clustered together while the most similar genomes from databases were from Senegal, Subsaharan Africa or other countries. Present data describes the circulation of IABV during the GMT over time in the population studied here shedding light on the viral dynamics in such setting of mass gathering.

## 1 Introduction

Infections with influenza viruses are among the most common respiratory viral infections and among those causing the most morbidity and mortality [1, 2]. Its epidemiology, diagnosis, and clinical consequences have been somewhat overshadowed by the SARS‐CoV‐2 pandemic, which has attracted a great deal of attention, interest, and research since its occurrence in 2020 [3]. Moreover, influenza viruses hardly circulated in many places during the winter of 2020–2021, for reasons that can be partially explained by the measures taken to prevent the transmission of SARS‐CoV‐2 infection but that are not fully explained, considering that other respiratory viruses continued to circulate during this period of time [4]. Also, obviously, genomic surveillance of influenza viruses, which was among the most active, has been far surpassed by that of SARS‐CoV‐2, with more than 18 million genomes available in global sequence libraries such as in GISAID [5], compared to 492,641 for influenza A/H3N2 (221,479), A/H1N1 (172,179), and B (98,983) viruses in GISAID [6]. and 425,796 for influenza A/H3N2 (129,634), A/H1N1 (116,116), and B (180,046) viruses in GenBank [7].

Genomic surveillance of influenza viruses is nonetheless of considerable importance, especially since it is one of the three groups of viruses for which there is a vaccine and treatment, along with SARS‐CoV‐2 and respiratory syncytial viruses A and B. The state of genomic data for IABV is heterogeneous worldwide. In particular, there are a relatively small number of genomes for Africa, with very little or no data for some countries [8]. This is unfortunate, as it remains poorly understood why influenza viruses, whose incidence is described as influenced by cold weather, circulates in Africa with a different epidemiology than that extensively studied in temperate countries [8]. Large population gatherings such as those during the Hajj in Mecca or the Grand Magal of Touba (GMT) provide considerable opportunities for infectious diseases to circulate globally, being transmitted between pilgrims after their importation by pilgrims from the countries where they live and being exported to these countries upon their return [9]. This has been widely demonstrated for various types of infections, particularly respiratory and gastrointestinal infections [9]. The GMT commemorates the departure into exile of Cheikh Ahmadou Bamba, founder of Mouridism, to Gabon in 1895. It is celebrated every 18th day of the month of Safar (Muslim calendar) in the city of Touba, Senegal. It attracted 6,583,278 individuals people from all over Senegal and elsewhere in 2025 [10]. The event is the largest religious gathering in West Africa, and, like any large mass gathering, it presents a risk of transmission of infectious diseases, mainly respiratory tract infections [11]. Here, we sought to obtain and analyze the genomes of IABV between 2017 and 2024 from individuals with respiratory infections sampled during the Grand Magal of Touba over seven consecutive years.

## 2 Materials and Methods

### 2.1 Population Studied

The GMT is celebrated every 18th day of the month of Safar (Muslim calendar), corresponding to October 28, 2018, October 17, 2019, October 6, 2020, September 26, 2021, September 15, 2022, September 4, 2023, and August 23, 2024. The study involved (i) cohorts of pilgrims who were sampled between 2018 and 2024 and departed from the villages of Dielmo (13°43′ 22.07″ N, 16°24′40.09″ W) and Ndiop (13°41′08.01″ N, 16°23′01. 01″ W) located in southern Senegal, in the Fatick region and (ii) patients who were sampled between 2018 and 2024 when consulting for respiratory tract infection at the Mbacké health care center (14°47′56″ N, 15°54′36″ W) located 10 km from Touba. The Mbacké health care center was chosen because of its high attendance by pilgrims during the 5 days of free GMT medical coverage (for instance, 1115 patients were managed during the 2015 GMT (unpublished data). Pilgrims from Ndiop and Dielmo involved in the present study were identified by the nurses in charge of the primary care centers in the two villages and participated in the study on a voluntary basis. Participants (or their parents if they were minors) signed a written consent form.

### 2.2 Data and Respiratory Sample Collection

For Dielmo and Ndiop pilgrims, at the time of inclusion, the procedure was to collect data using a standardized questionnaire 7 to 10 days before departure to Touba and 6 to 9 days after return. The survey covered demographic data (age and gender), chronic diseases, respiratory symptoms and hospitalization rates. Respiratory sample collection was systematically proposed before and after participation to the GMT. At the Mbacké health center, during the 5 days of GMT with free medical coverage, the medical team completed a demographic and clinical questionnaire, and patients were proposed respiratory sampling. Nasopharyngeal samples were taken using commercial rigid cotton swab applicators inserted into the anterior nares and oropharynx, then placed in a viral transport medium (Virocult Sigma, Burlington, MA, USA), as appropriate for further PCR analyzes. Samples were kept at +4°C (range, 2°C–8°C) before being transported to the Dakar laboratory for storage in a –80°C freezer, then transferred to Marseille on dry ice for processing.

### 2.3 qPCR Testing for Influenza A/B Viruses

Nucleic acid extraction was performed from the samples using the MagMAX Viral/Pathogen Nucleic Acid Isolation Kit on the KingFisher Flex system (Thermo Fisher Scientific, Waltham, MA, USA). Real‐time reverse transcription (RT)‐PCR (qPCR) for IABV was performed using the Multiplex RNA Virus Master Kit (Roche Diagnostics, Meylan, France) on a C1000 Touch thermal cycler (Bio‐rad, Hercules, CA, USA). The sequences of primers and probes that targeted influenza A/B viruses are listed in Supporting Information Table S1. Negative controls (RNase‐free water) and positive controls (extracted RNA from Influenza A and B culture supernatants) were included in all series.

### 2.4 Next‐Generation Sequencing and Analysis of Influenza A/B Virus Genomes

PCR amplification of IAV virus was performed as previously described [12]. using a previously set up protocol that specifically targets the 5′ and 3′ hyperconserved regions of all eight genomic segments from all animal (including human) IAV viruses [13, 14]. PCR amplification of IBV virus was performed using another previously described protocol [15, 16]. PCR amplicons were sequenced using Illumina technology on a NovaSeq. 6000 instrument (Illumina, San Diego, CA, USA) with the paired‐end strategy and the COVIDSeq protocol (Illumina Inc.).

IABV virus genomes were assembled from NGS reads following a previously described procedure [12] using Dragen Bcl Convert pipeline v3.9.3 (Illumina Inc.) [17], trimmomatic (v. 0.39) [18], minimap2 (v2.17‐r941) [19], samtools (v. 1.14) [20], FreeBayes v1.3.5 [21], and sam2consensus (v2.0) [20] (Supporting Material). For phylogeny, sequences were aligned using the Mafft software v7.505 [22]. Then, phylogenetic trees were constructed by IQTREE v2.2.0 [23] and visualized and with ITOL [24]. Finally, clade, sub‐clade as well as amino acid mutations were identified using the Nexclade software [25, 26, 27]. Viral classification was carried out on genome segments 4 and 6, which encode the hemagglutinin (HA) and the neuraminidase (NA), respectively. The two nucleotide sequences most similar to each of the sequences obtained here were collected from the GISAID nucleotide sequence database, EpiFlu database (GISAID EpiFlu Database) [6], and were incorporated in the phylogeny reconstruction, using MEGA v 6.4 (https://www.megasoftware.net/) and MrBayes V.3.2. (20). Viral reassortants were searched based on these trees using GiRAF [28], [https://github.com/Kingsford-Group/GIRAF]. Antigenic diversity analysis used PREDAV‐FluA [29, 30].

## 3 Results

### 3.1 Epidemiological Characteristics of the Studied Population

The study included 1217 participants with 727 pilgrims who lived in the villages of Dielmo or Ndiop and 490 patients who consulted at the Mbacké health center for respiratory tract infection symptoms (Table 1). The 727 pilgrims comprised 47% of men. Their mean age was 25 years (range, 0–85 years). A total of 30 (4.1%) pilgrims reported underlying chronic diseases, with high blood pressure being the most common. Besides, the 490 patients consulting at the Mbacké health center comprised 41% of men and their mean age was 18 years (range, 0–80 years).

**Table 1:** Demographic, clinical characteristics and qPCR results.

Features	Pilgrims for Ndiop and Dielmo (all) (*N* = 727)	Pilgrims for Ndiop and Dielmo with influenza virus (*N* = 26)	Mbacké patients consulting for respiratory symptoms (all patients) (*N* = 490)	Mbacké patients with influenza virus (*N* = 177)
Mean age (years)	25.1	19.8	18.2	17.7
Sex (male)	340 (46.8%)	17 (65.4%)	202 (41.2%)	78 (44.06%)
Chronic diseases	30 (4.1%)	0 (0.0%)	n.d.	n.d.
Respiratory symptoms	385 (52.9%)	20 (76.9%)	490 (100%)	177 (100%)
Cough	282 (38.8%)	17 (65.4%)	490 (100%)	177 (100%)
Dyspnea	82 (11.3%)	2 (7.7%)	14 (2.9%)	1 (0.6%)
Rhinitis	294 (40.4%)	16 (61.5%)	338 (69%)	33 (18.6%)
Sore throat	117 (16.1%)	7 (26.9%)	95 (19.4%)	97 (54.8%)
Fever	147 (20.2%)	13 (50.0%)	207 (42.2%)	90 (50.8%)
Positive PCR for influenza virus	26 (3.6%)	26 (100.0%)	177 (36.1%)	177 (100.0%)
Successfull sequencing	—	23/26 (88.5%)	—	167 (94.4%)

Abbreviation: n.d., not documented.

### 3.2 Clinical Characteristics of the Studied Population

Of the 727 pilgrims from the villages of Dielmo or Ndiop, 385 (53%) had respiratory symptoms during the study period and 142 (19.5%) reported fever. The most common respiratory symptoms were rhinitis, cough, and sore throat. Regarding the 490 patients who were included at the Mbacké health center, all had a cough, 207 (42.2%) had fever, and the other most common respiratory symptoms were rhinitis and sore‐throat (Table 1).

### 3.3 Results of qPCR Testing for Influenza A and B Viruses

Overall, 203/1,217 individuals (16.7%) tested positive for IABV by qPCR including 173/203 (85.2%) for IAV and 30/203 (14.8%) for IBV. The overall prevalence of IABV in pilgrims from Ndiop and Dielmo was 3.6% (26/727) including 6/26 (23.1%) pilgrims without respiratory symptoms. Most positive pilgrims (23/26, 88.5%) had IAV infection and the remaining IBV infection (3/26, 11.5%). The prevalence of IABV infection in patients with respiratory symptoms at the Macké health center was 36.1% (177/490) with 150/177 (84.7%) cases due to IAV and 27/177 (15.3%) cases due to IBV.

**Figure 1:**
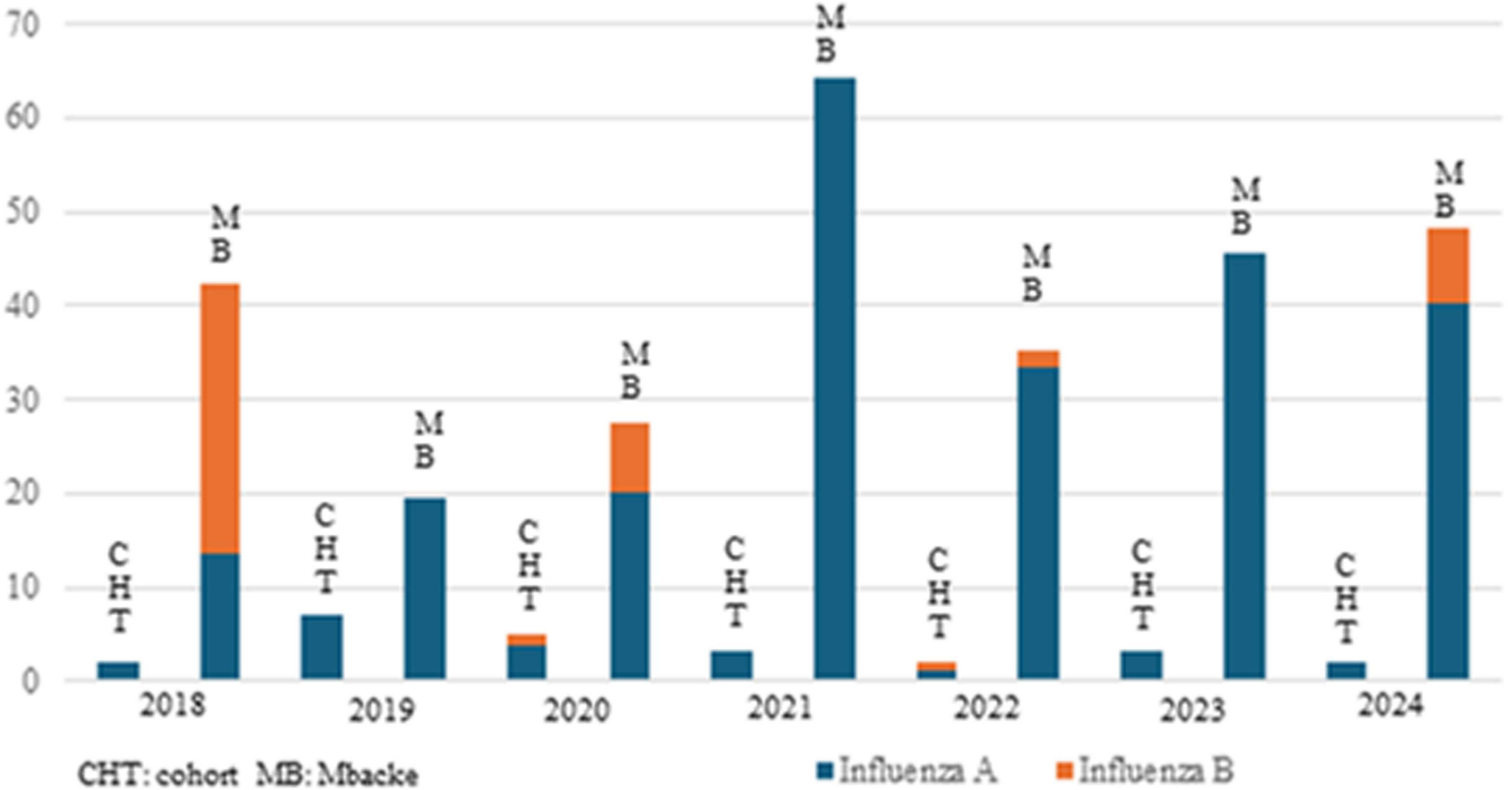
Distribution of influenza A/B cases (2018–2024).

The yearly distribution of positive rates is presented in Figure 1. IAV was detected throughout the study period but it was the most prevalent in 2021 (64.2%) and 2023 (45.5%) among patients consulting at the Mbacké health care center. In contrast, IBV was the most prevalent in 2018 (28.8%) among patients in Mbacké and was not detected in 2019, 2021, and 2023.

### 3.4 Influenza A and B Virus Genome Analyzes

**Figure 2:**
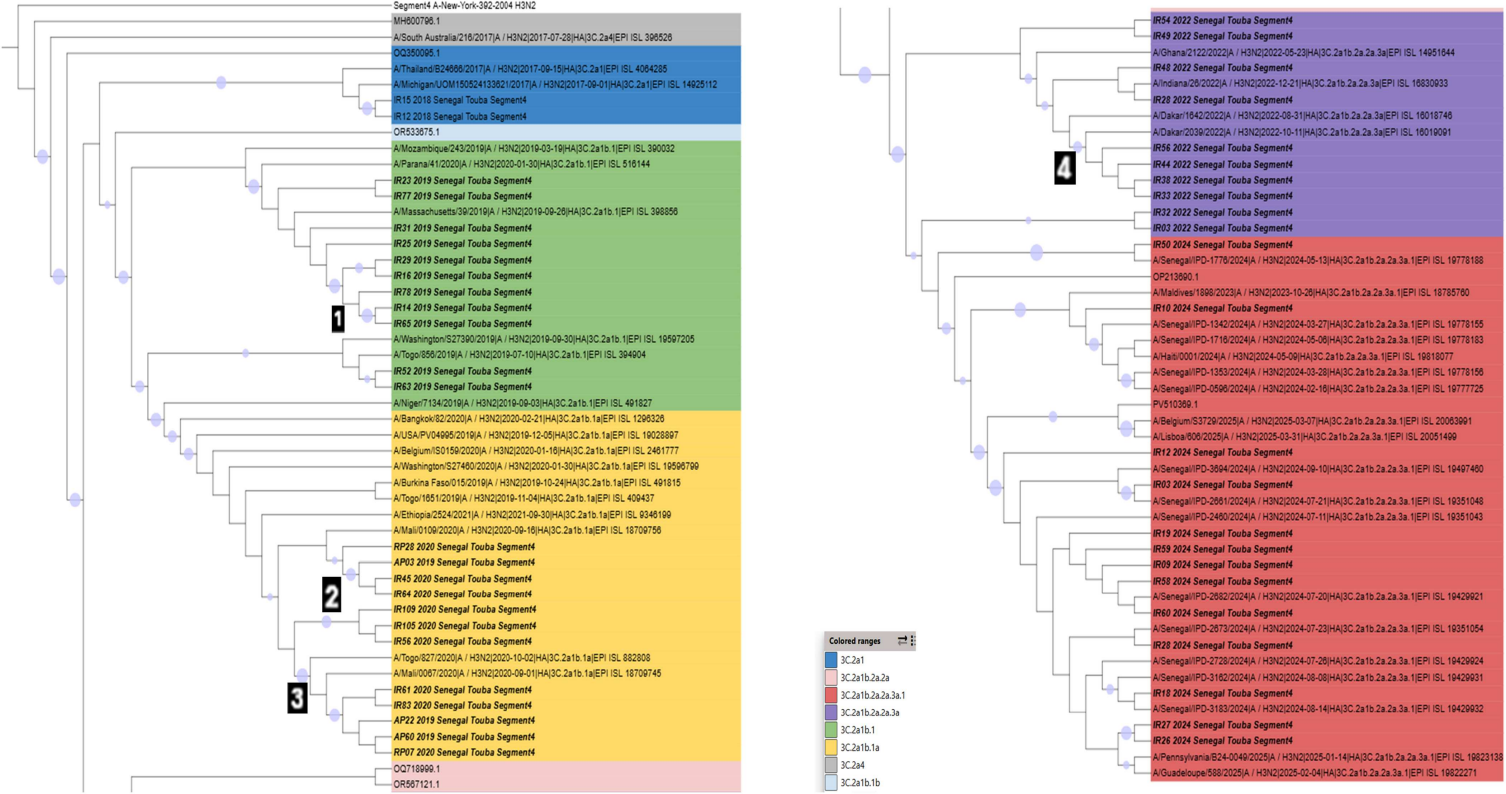
Phylogenetic tree based on the segment 4 from the influenza A/H3N2 virus genomes. Only boostrap values greater than or equal to 70 were represented, indicated by a pale blue circle. Clusters 1–4 are indicated by a white font and a black background and are quoted in the text.

**Figure 3:**
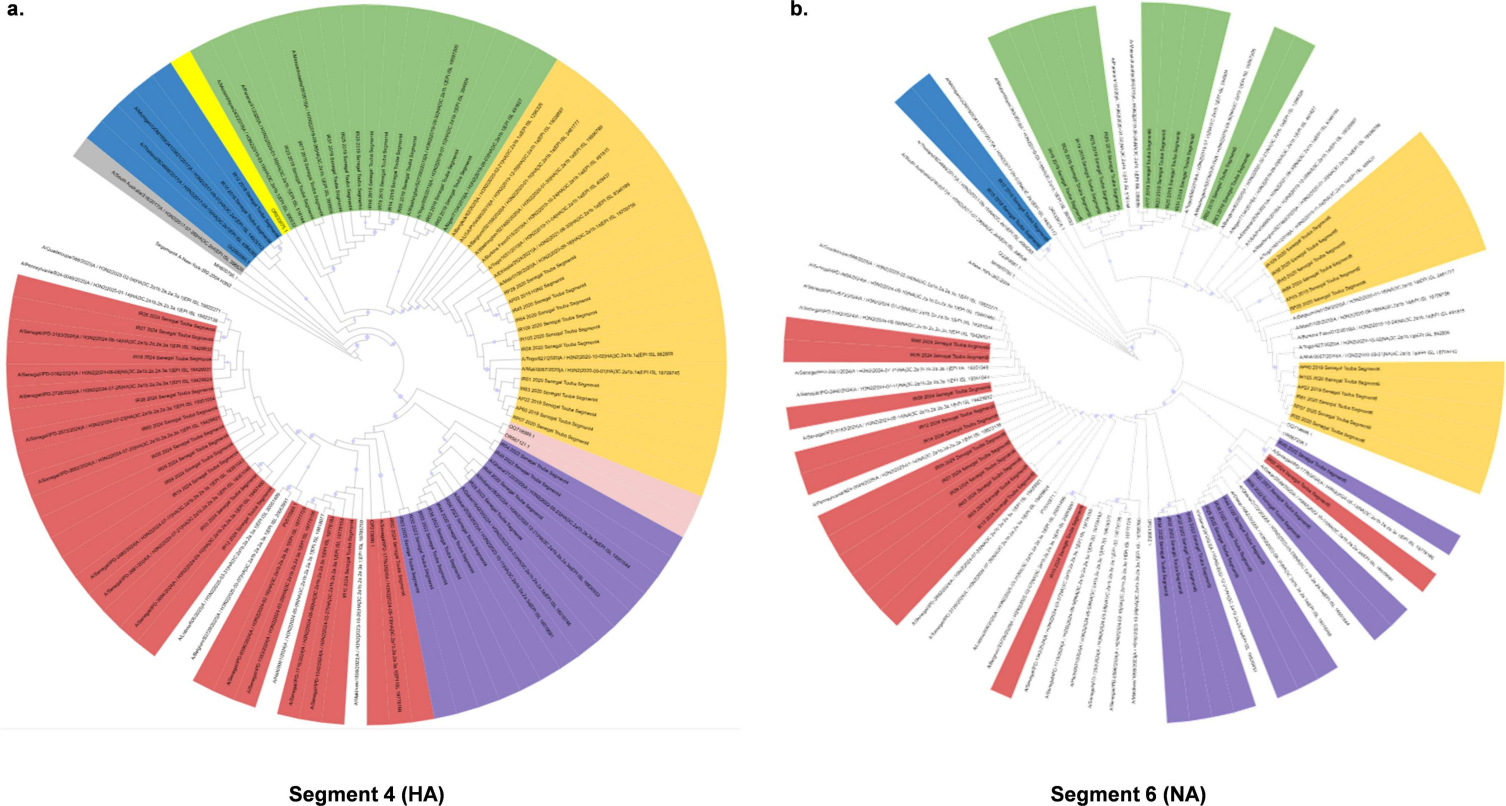
Phylogenetic tree based on the segment 4 and 6 from the influenza A/H3N2 virus genomes. Only boostrap values greater than or equal to 70 were represented, indicated by a pale blue circle.

NGS of IAV or IBV was performed for all 190 nasopharyngeal samples that were found as positive by qPCR. After quality control and assembly of NGS reads, 106 (55.8%) complete genomes were obtained with all eight segments fully‐covered. Of them, 99 (93.4%) corresponded to IAV and seven (6.6%) to IBV. Viral genomes were recovered every year with 4 (3.8%), 14 (13.2%), 16 (15.1%), 27 (25.5%), 10 (9.4%), 22 (20.7%), and 13 (12.3%) IAV or IBV genomes obtained in 2018, 2019, 2020, 2021, 2022, 2023 and 2024, respectively. Regarding IAV, genomes were obtained from 99 samples collected between 2018 and 2024. Fourty‐eight (48.5%) were classified as of the A/H3N2 subtype and 51 (51.5%) as of the A/H1N1 subtype, based on the genomic segments encoding HA and NA. As for the A/H3N2 genomes, 2 (4.2%), 14 (29.1%), 9 (18.8%), 10 (20.8%), and 13 (27.1%), were obtained in 2018, 2019, 2020, 2022, and 2024, respectively. According to phylogenetic analysis based on segment 4 (that encodes the HA), sequences obtained here were classified into five main clades that changed over the years (Figure 2). For 2018, all two sequences (4% of all A/H3N2 genomes) were classified as of clade 3 C.2a1 and subclade B.1. For 2019, all 11 sequences (23%) were classified as of clade 3 C.2a1b.1 and subclade B.1.2.1. For 2020, all 12 sequences (25%) were classified as of clade 3 C.2a1b.1a and subclade B.1.2.1.1. For 2022, all 10 sequences (21%) were classified as of clade 3 C.2a1b.2a.2a.3a and subclade G.1.3.1. For 2024, all 13 sequences (27%) were classified as of clade 3 C.2a1b.2a.2a.3a.1 and within three subclades including J.2.2 in 11 cases and J.1.1 and J.4 in one case each; none of the 23 IAV genomes obtained for 2023 were classified as A/H3N2. Based on the analysis carried out with segment 6 (that encodes the NA), the sequences were classified into three clades including A.2.2 in 25 cases (52% of all A/H3N2 genomes) in 2018, 2019, and 2020; B.4 in 11 cases (23%) in 2022; and B.4.2 in 12 cases (25%) in 2024 (Figure 3). Considering segment 4, some sequences from the GMT were in clusters (see clusters 1–4 in Figure 2), apart from other sequences from Senegal or elsewhere that were nonetheless their best BLAST hits. Overall, mean (± standard deviation) nucleotide identity between GMT sequences was 97.77% ± 1.39% (range, 95.70%–100.00%).

A total of 42 different sequences were retrieved from the GISAID database as those two the most similar to each sequence obtained here. They were obtained over the period from July 2017 to March 2025 and from various African countries including Senegal, Niger, Togo, Mali, Mozambique, Burkina Faso, Ghana, and Ethiopia, as well from other countries outside this continent, such as the USA, Thailand, Belgium, Brazil, Guadeloupe, and Portugal. Mean pairwise amino acid similarity between the sequences obtained here and their two best matches was 97% ± 0.02% (range: 93.8%‐100%) for HA and 98 ± 0.01% (range: 91.7%–100%) for NA. Besides, mean pairwise amino acid similarity was 94% ± 0.03% (90%–100%) for HA sequences and 96 ± 0.02 (range: 91%–100%) for the NA sequences obtained here with the sequences of the A/H3N2 vaccine strains used in the southern hemisphere, and mean amino acid difference by year was 4.3% in 2018, 5.5% in 2019, 6.6% in 2020, 1.6% in 2022, and 3.1% in 2024 for hemagglutinin, and 2.1% in 2018, 2.6% in 2019, 2.9% in 2020, 1.1% in 2022, and 1.3% in 2024 for neuraminidase. Overall, each GMT was associated with the detection of an A/H3N2 clade and one or more subclades that had not been detected in previous or subsequent years, with the exception of three A/H3N2 genomes from 2019 that were grouped with genomes from 2020 (Figure 1). Regarding the comparative analysis with the most similar sequences, one sequence from a sample collected in December 2017 in Switzerland felt within the 2018 cluster, and three sequences from samples collected in December 2012 in France and one sequence from a sample collected in January 2024 in England felt in the 2023 cluster.

**Figure 4:**
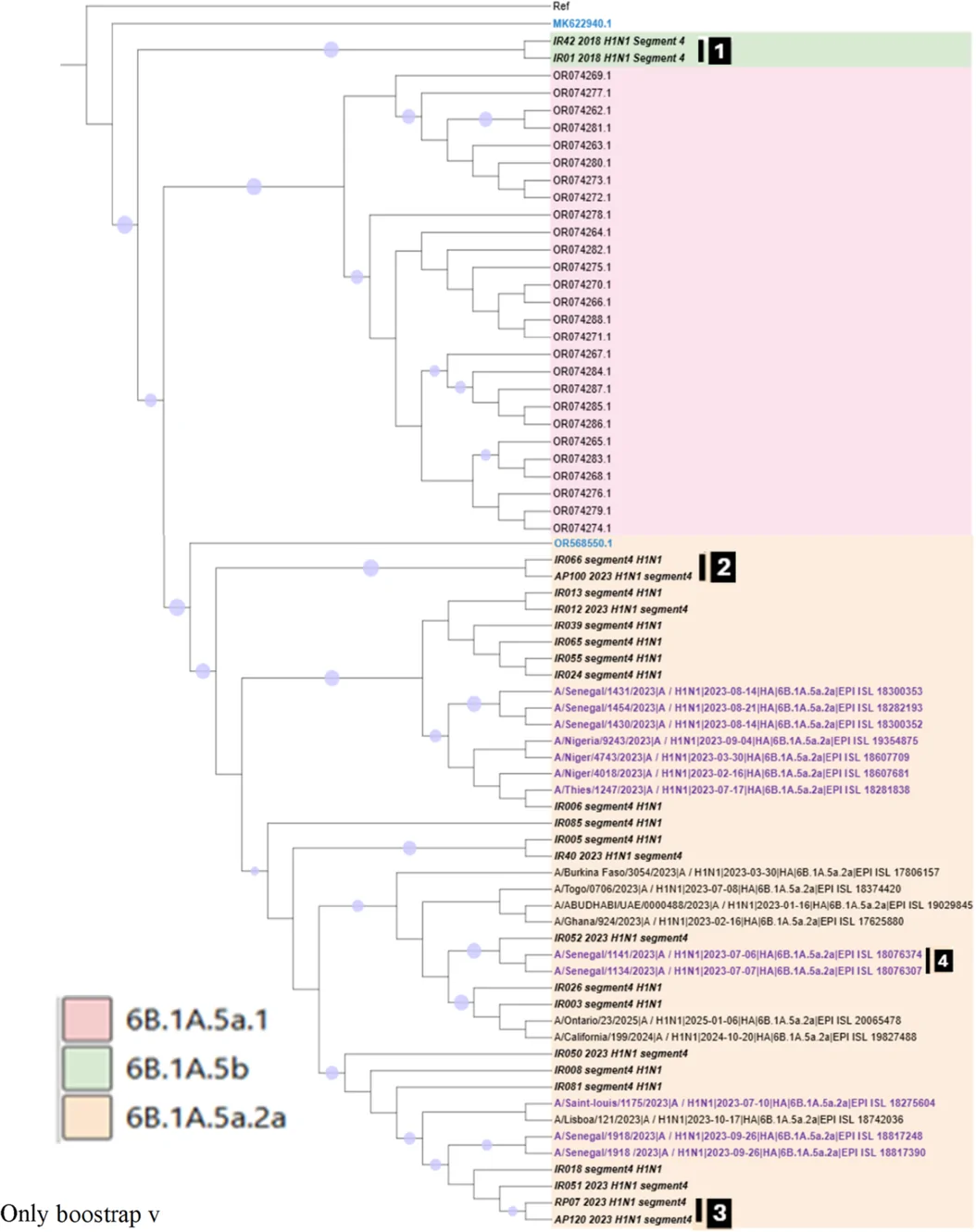
Phylogenetic tree based on the segment 4 from the influenza A/H1N1 virus genomes. Only boostrap values greater than or equal to 70 were represented. Clusters 1–4 are indicated by a white font and a black background and are quoted in the text.

**Figure 5:**
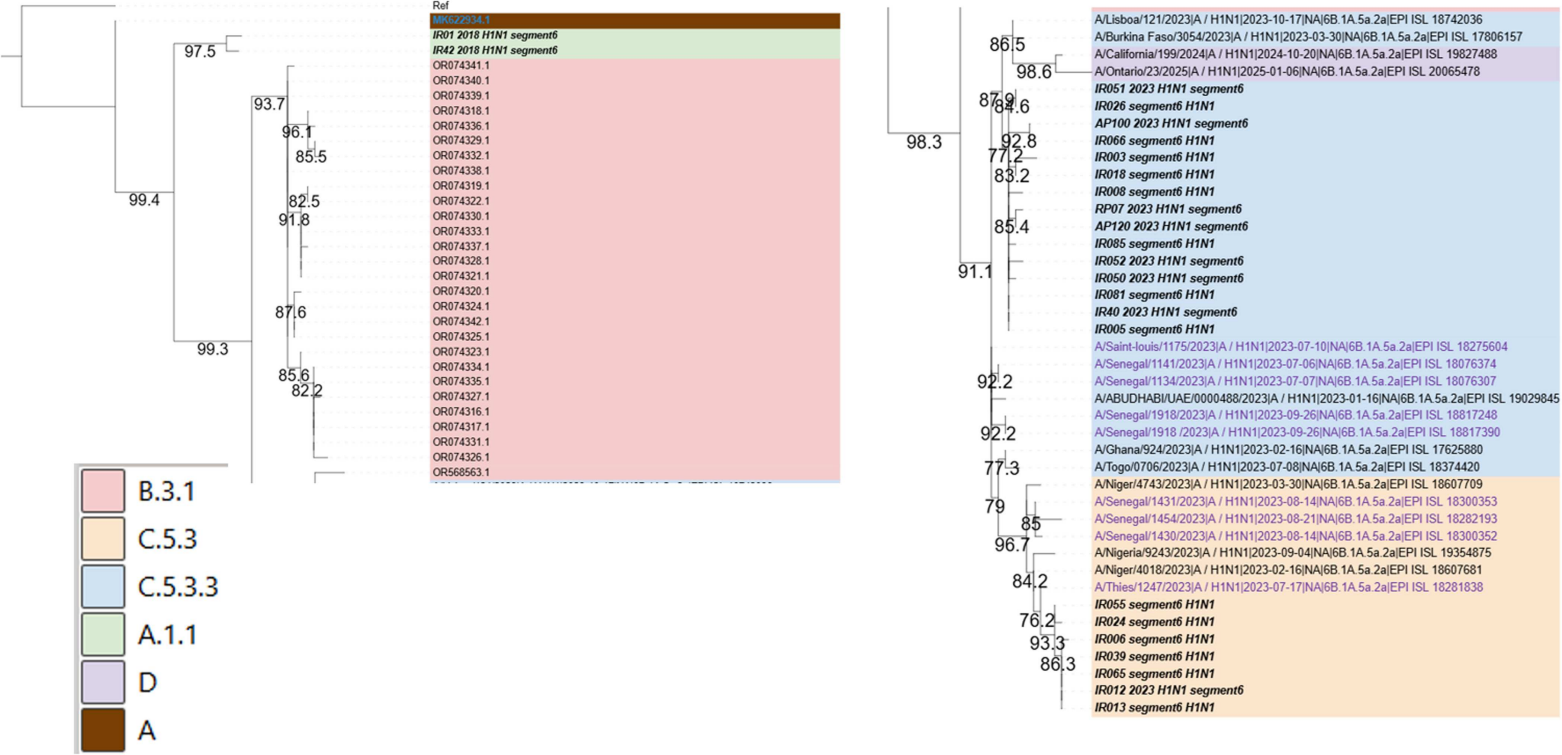
Phylogenetic tree based on the segment 6 from the influenza A/H1N1 virus genomes. Only boostrap values greater than or equal to 70 were represented.

As for the A/H1N1 genomes, two (3.9%), 27 (52.9%) and 22 (43.1%) genomes were obtained in 2018, 2021 and 2023, respectively. According to the phylogenetic analysis based on segment 4, sequences obtained here were classified into clade 6B.1 A.5b and subclade 5b for 2018, into clade 6B.1 A.5a.1 and subclade B for 2021, and into clade 6B.1 A.5a.2a and subclade C.1 (21 sequences) and C.1.9.1 (1) for 2023 (Figure 4). Based on the analysis carried out with segment 6, sequences were classified into three clades including A.1.1 (two (3.9%) sequences for 2018), B.3.1 (27 (52.9%) sequences for 2021), C.5.3 (15 (31.9%) sequences for 2023), and C.5.3.3 (7 (14.9%) sequences for 2023) (Figure 5). Considering segment 4, some sequences from the GMT were in clusters (see clusters 1–3 in Figure 4), apart from other sequences from Senegal or elsewhere that were nonetheless their best BLAST hits. Overall, mean (± standard deviation) nucleotide identity between GMT sequences was 99.01% ± 0.81% (range, 96.90%–100.00%). Nucleotide identity was 99.90% between the two sequences of cluster 1, and 100.00% between the two sequences of clusters 2 and 3. A total of 19 different sequences were retrieved from the GISAID database as those two the most similar to each sequence obtained here. They covered the period from December 2022 to January 2024 and were mainly obtained from Senegal (9 sequences), then from Niger (2), and from the USA, Nigeria, Ghana, Togo, Burkina, Portugal, Canada, and United Arab Emirates (one sequence each). Mean pairwise similarity of amino acids between the sequences obtained here and their two best matches was 98.0% ± 0.01% (range: 95%–100%) for HA and 91% ± 0.06% (range: 80%–100%) for NA. Besides, mean pairwise amino acid similarity was 89.0% ± 0.06 (range: 79%–100%) for HA sequences and 93 ± 0.05 (range: 84%–100%) for the NA sequences obtained here with the sequences of the A/H1N1 vaccine strains used in the southern hemisphere. The amino acid difference compared to these vaccine strains was 6.4% for 2018% and 5.3% for 2023, and the GMT was associated with the detection of an A/H1N1 clade and one or more subclades that had not been detected in previous or subsequent years. Genomes obtained for year 2021 and described in a previous study by the same team [12] followed the same pattern, as they comprised a single cluster, apart from any other sequences analyzed here.

**Figure 6:**
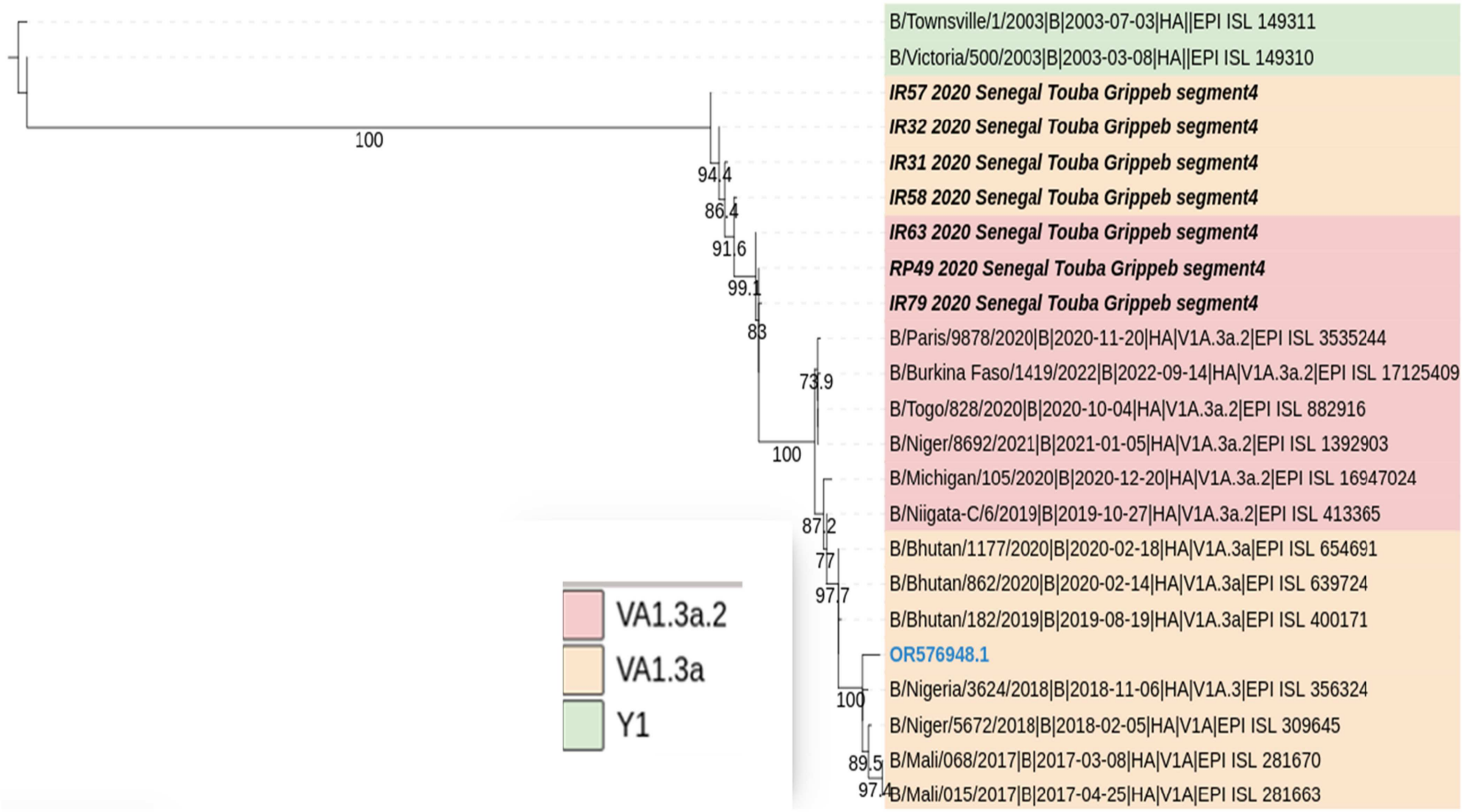
Phylogenetic tree based on the segment 4 from the influenza B virus genomes. Only boostrap values greater than or equal to 70 were represented.

**Figure 7:**
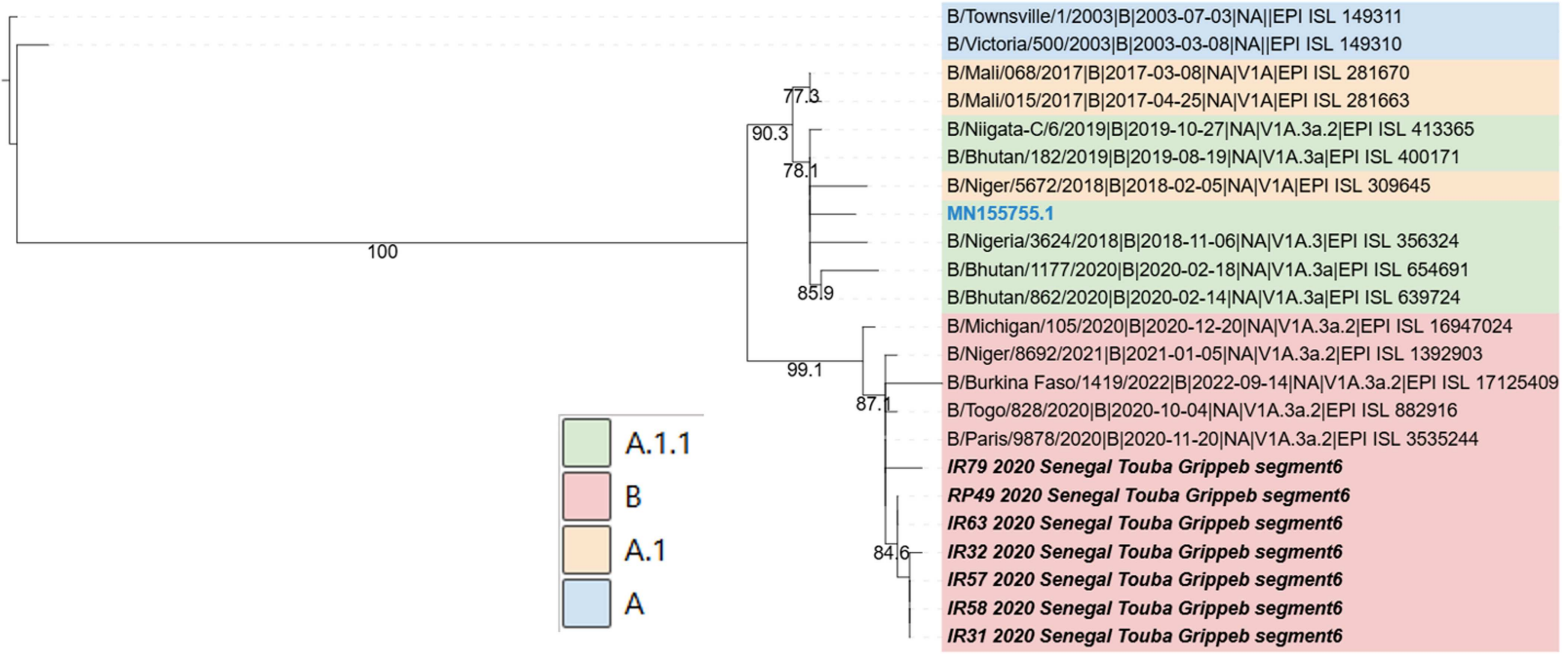
Phylogenetic tree based on the segment 6 from the influenza B virus genomes. Only boostrap values greater than or equal to 70 were represented.

As for IBV, the seven complete genomes obtained here were all from 2020. Based on segment 4, four sequences belonged to clade V1A.3a, subclade A.3.1, while the other three belonged to clade V1A.3a.2 (Figure 6). Based on segment 6, all sequences belonged to clade B (Figure 7). Fifteen different sequences were retrieved from the GISAID database as those two the most similar to each sequence obtained here. They covered the period from March 2017 to August 2023 and were obtained from the USA (3 sequences), Bhutan (3), Mali (2), Niger (2), and France, Togo, Nigeria, Burkina Faso and Japan (one sequence each). Mean pairwise amino acid similarity was 96% ± 0.03% (range, 88%–100%) between HA sequences and 93 ± 0.05 (range, 82%–100%) for NA sequences obtained here and their two best hits was. It was 93 ± 0.03 (range, 92%–100%) for HA and 98 ± 0.03 (range, 91%–100%) for NA with sequences from B vaccine strains used in the southern hemisphere. Besides, amino acid differences versus vaccine strains were 7.0% for HA and 2.0% for NA.

Finally, among all our A/H3N2 and influenza B sequences, no antigenic divergence was observed compared to the respective vaccine strains for the same period. Regarding A/H1N1strains, all strains in clade C.1 showed no divergence from the vaccine strain. However, the A/H1N1 sequence from clade C.1.9.1 showed divergence from the vaccine strain, with genetic mutations noted at positions G11 A, G35A, C175T, G208A, and G260A. This suggests an antigenic change, making this strain different from the vaccine strain. As for potential inter‐clade reassortment, none was detected based on genome segments obtained here. Additional data on amino acid substitutions are available in the Supporting Material.

## 4 Discussion

Here, we show the emergence and evolution of A/H3N2 and A/H1N1 lineages over a 7 year period. The arrival of SARS‐CoV‐2 has had a major impact on influenza epidemics worldwide. In this study, we analyzed 94 complete genomes of IAV (A/H1N1, A/H3N2) and IBV genomes over a 7‐year period (2018–2024) obtained from patients with respiratory symptoms during the GMT in Senegal, one of the largest religious gatherings in the country. This period coincides in part with the SARS‐CoV‐2 pandemic. We identified different clades and subclades of influenza viruses that circulated in the pilgrims included in the present study between 2018 and 2024. These clades and subclades were determined based on segments 4 and 6 of the viral genome, encoding hemagglutinin (HA) and neuraminidase (NA), respectively. In recent years, three subtypes of IAV (A/H1N1, A/H2N2, and A/H3N2) have been circulating in the human population. These viruses were the cause of epidemics against which humans lacked neutralizing antibodies, resulting in fulminant worldwide spread, with millions of infections and increased mortality [31]

In our study, the A/H1N1 subtype was only detected in 2018, 2021, and 2023. The sequences from 2018 belonged to clade 6B.1 A.5a, those from 2021 to clade 6B.1A.5a.1 [12], and those from 2023 to clade 6B.1A.5a.2a. The A/H3N2 subtype was identified for the years 2018, 2019, 2020, 2022, and 2024, with each year corresponding to a different clade. In fact, the A/H3N2 viruses detected each belonged to a different clade, indicating genetic evolution and diversity of circulating strains over the years. We identified seven complete influenza B genomes, all from 2020.

Based on the GISAID database, 391 genomes (2406 sequences) of A/H1N1, 316 genomes (1774 sequences) of A/H3N2, and 217 genomes (1064 sequences) of influenza B are available for Senegal [6]. Compared to previous data on influenza in Senegal, a study published in 2012 reported 77, 272, and 60 sequences of the HA and NA genes for viruses A(H1N1), A(H3N2), and B, respectively, over the period 2008–2009 [32]. A subregional study on influenza involving four countries Senegal, Cape Verde, Mauritania, and Guinea for the years 2009 and 2010 shows that out of 3155 samples, 503 sequences were classified as A/H1N1, 314 as A/H3N2, and 85 as influenza B [33]. In addition, a multicenter study conducted in nine European countries by Kissling et al. for the 2021–2022 season showed that out of a total of 153 samples, 53 were sequenced and all belonged to clade 6B.1A.5a.1 [34]. Another study by Melidou et al. covering the 2021–2022 season analyzed a total of 38 A/H1N1 viruses, 24 of which belonged to clade 6B.1 A.5a.1 [35]. IABV nucleotide and amino acid mutations that were detected here could be further studied to assess their functional impact including on virus transmissibility or pathogenicity, which could be conducted using in silico structural predictions and in vitro phenotypic assays, and should be confirmed through larger epidemiological studies.

An interesting finding is the clustering and high pairwise sequence similarities for sequences obtained here from samples collected during the same GMT or two consecutive GMT. This could suggest the existence of viral transmission chains during this event, or as for the cases of 2019 and 2020, of the circulation of a same clade during two consecutive years. A limitation to the interpretation of these findings is the limited number of available genomes and sequences for Senegal and its geographical area over the study period and therefore these clustering might simply reflect the detection of dominant strains already prevalent across Senegal or West Africa. Besides, our study coincided in part with the SARS‐CoV‐2 pandemic. Before the pandemic (January 2013–March 2020), the epidemiology of influenza in Senegal was characterized by continuous circulation of the virus throughout the year, with two distinct seasonal peaks, the first expected from January to March and the second during the rainy season in August. With the arrival of SARS‐CoV‐2 in Senegal (March 2020–July 2022), only the influenza peak expected from January to March was affected, but the main peaks of the rainy season remained unchanged in their timing and intensity [36]. Nevertheless, the effect of the COVID‐19 pandemic on the epidemiology of influenza virus infections in Senegal makes therefore difficult comparing influenza virus data during the normal periods and during the pandemic period.

Our study has several limitations including notably its relatively small sample size which is due to the design of our survey that was limited to small groups of pilgrims originating from two villages in South Senegal where our group has implemented surveillance projects since decades and to pilgrims consulting at one health care center near Touba that was chosen for its high activity during the GMT. Also, our studies did not take place during the peak activity of influenza in Senegal but were conducted at the time the GMT took place, leading to a 16.7% positivity rate for IABV by qPCR. Finally, only 55.8% of positive samples could be analyzed by sequencing methods due to low viral loads in many participants. In addition, the study population comprised two heterogeneous groups with different baseline health statuses, exposure risks, and healthcare‐seeking behaviors, which may obscure the true infection risks and transmission dynamics. Furthermore, the pilgrims from Dielmo and Ndiop are not representative of all pilgrims participating to the GMT. For all these reasons, generalization of our results to all GMT pilgrims is not possible. Also, due to its design, our study lacks surveillance data from the period immediately preceding the GMT to serve as a background, making it difficult to assess the extent to which the GMT itself amplified the inherent seasonal peaks.

Overall, these data, and their comparison with those in the literature and sequence libraries for Senegal, highlight a low level of genomic surveillance compared to what has been implemented for SARS‐CoV‐2. A broader phylogenomic study in other contexts than the GMT could help to better understand how influenza viruses circulate and spread in geographical areas such as Senegal, where there is no cold season. Overall, the GMT represents a remarkable opportunity to better describe and understand the circulation of influenza viruses globally through such phylogenomic studies.

## Author Contributions

Conceived and designed the experiments: Cheikh Sokhna, Philippe Gautret, Philippe Colson. Contributed materials, analysis tools: All authors. Analyzed the data: Safietou Diallo, Coumba Diouf, Mamadou Beye. Writing—original draft preparation: Coumba Diouf, Safietou Diallo. Writing—review and editing: All authors. All authors have read and agreed to the published version of the article.

## Ethics Statement

The protocol was approved by the National Ethics Committee for Health Research in Senegal (SEN17/62) and performed in accordance with the good clinical practices recommended by the Declaration of Helsinki and its amendments.

## Conflicts of Interest

Philippe Colson is a scientific advisor of BioSellal and Triber companies. Other authors have no conflicts of interest to declare. Funding sources had no role in the design and conduct of the study; collection, management, analysis, and interpretation of the data; preparation, review, or approval of the article.

## Supporting information


Supporting File 1

Supporting File 2

## Data Availability

The data that support the findings of this study are available on request from the corresponding author. The data are not publicly available due to privacy or ethical restrictions. IABV genomes analyzed here have been submitted to the GenBank sequence database (https://www.ncbi.nlm.nih.gov/genbank/) with GenBank Accession no. PX430499‐PX430554, PX414631‐PX415195, and PX457773‐PX457783.
