# Supplementary material for: Epidemiological, Clinical and Viral Genomic Features of Influenza Virus Infections Among Pilgrims During the Grand Magal of Touba Over 7 Years (2018–2024)
Source: J Med Virol. 2026 Aug 24;98(8):e71120. doi: 10.1002/jmv.71120 (PMC13501545; doi:10.1002/jmv.71120)
Supplement: Supplementary file 1 [file JMV-98-e71120-s001.docx]

**SUPPLEMENTARY MATERIAL**

**Supplementary methods**

**Next-generation sequencing and analysis of influenza A/B virus genomes**

The « tagmentation » step fragmented and tagged the DNA. Then limited PCR amplification (12 cycles) completed the tag adapters and introduced dual-index barcodes. After purification on ITB beads (Illumina Inc.), the libraries were normalized to the same molarity and pooled into a single library that was denatured. Then, automated cluster generation and paired-end sequencing with dual index reads were performed, and loading on a S1 flow cell and the NovaSeq 6000 instrument (Illumina Inc.) was carried out with a reading of 2x150 base pairs, according to the NovaSeq-XP workflow (Illumina Inc.) and following a previously described procedure [16].

IABV virus genomes were assembled from NGS reads following a previously described procedure [12]. Briefly, base calling was carried out with the Dragen Bcl Convert pipeline v3.9.3 (Illumina Inc.) [17]. Quality control and adaptor trimming of raw reads was performed with trimmomatic (v. 0.39) [18]. Thereafter, trimmed reads were aligned using minimap2 (v2.17-r941) [19] against reference genomes from four IAV strains (namely IAV A/California/07/2009(H1N1), IAV A/Shanghai/02/2013(H7N9), IAV A/Korea/426/1968(H2N2) and IAV A/New York/392/2004(H3N2), as well as from three IBV strains (namely, IBV Victoria B/Brisbane/60/2008-egg, Yamagata B/Wisconsin/01/2010 and Yamagata B/Fukushima/H151/2018). PCR primers were removed with samtools (v. 1.14) and SAM files of read mapping were converted into BAM files [20]. Finally, variant calling was performed with FreeBayes v1.3.5 [21], and consensus sequences for IABV virus genomes were generated for each sample using sam2consensus (v2.0) [20].

For phylogeny, sequences were aligned using the Mafft software v7.505 [22]. Then, phylogenetic trees were constructed by IQTREE v2.2.0 [23] using the Neighbor-joining method with a boostrap analysis based on 1,000 replicates and Maximum Likelihood as model, and they were visualized and annotated with ITOL [24]. Finally, the clade, sub-clade as well as amino acid mutations were identified using the Nexclade software [25, 26] with as reference genomes those from A/Wisconsin/588/2019 for influenza A/H3N2 virus, from A/Darwin/6/2021 for influenza A/H1N1 virus, and from IBV virus Victoria B/Brisbane/60/2008-egg and Yamagata B/Wisconsin/01/2010 for IBV. Regarding amino acid mutations, only those present in genome sequences obtained from at least five samples were taken into account. Viral classification was carried out on genome segments 4 and 6, which encode the hemagglutinin (HA) and the neuraminidase (NA), respectively. The two nucleotide sequences most similar to each of the sequences obtained here were collected from the GISAID nucleotide sequence database, EpiFlu database (GISAID EpiFlu Database) [6], and incorporated were in the phylogeny reconstruction. Alignments of nucleotide sequences obtained here and from the GISAID database were performed using ClustalW with BioEdit v.7.2.5 (https://bioedit.software.informer.com/), and pairwise nucleotide identities were calculated from the multiple alignment, taking into account all aligned positions including indels; a same procedure was applied on amino acid sequences obtained by translation with BioEditv.7.2.5. Finally, alignments of each viral genome segment were generated by MEGA v 6.4 (https://www.megasoftware.net/) with Muscle and run through the MrBayes V.3.2.6 software to generate trees with >200,000 generations with a sampling frequency of 200 (20). Viral reassortants were searched based on these trees using GiRAF that automatically detects reassortments in cases of phylogenetic inconsistency [27] with default parameters except for cull modified to 10%.

**Analysis of antigenic diversity**

To analyze the antigenicity of the virus, we used a model called PREDAV-FluA [28, 28]. This is a computer tool designed to predict antigenic variants of the IAV based on conserved antigenic structures. It is based on analysis of the HA1 protein sequence, a key component of HA. In our study, we applied this model to analyze the antigenicity of the viral strains studied. We retrieved the HA1 sequences of all our strains (A/H1N1, A/H3N2, and IBV) as well as those of the reference vaccine sequences. All HA1 sequences were aligned using Mafft v7.505 software before being submitted to the model.

**Supplementary results**

As for the A/H3N2 genomes, In our dataset, several amino acid substitutions were associated with specific clades of our A/H3N2 strains. In the HA-encoding gene, for clade 3C.2a1b.1a, the A138S substitution was present in all sequences, while R299K was detected in five sequences from this clade. For clade 3C.2a1b.1, substitutions D186G, N190D, and S193F were observed in all sequences, and also present in two sequences assigned to clade 3C.2a1. In addition, R261Q was detected in nine sequences belonging exclusively to clade 3C.2a1b.1. In clades 3C.2a1b.2a.2a.3a.1 and 3C.2a1b.2a.2a.3a, substitutions E50K, N96S, and I192F were present in all sequences. In clade 3C.2a1b.2a.2a.3a.1, the T65K, N122D, S124N, and K276E substitutions were detected in 11 sequences, while I140K was present in all sequences in this clade. In the NA-encoding gene, the R400K mutation was observed in all sequences of clade B.4.2, and I65V was observed in seven sequences of clade B.4. The R315S, D346G, and N463D substitutions were present in all sequences of clade A.2.2. S44F, K344E and V263I were observed in six, seven and twenty-three sequences of the same clade, respectively.

As for the A/H1N1 genomes, in our dataset, several amino acid substitutions were associated with specific clades of our A/H1N1 strains. In the HA-encoding gene, mutations K54Q, A186T, E224A, R259K, and K308R were present in all sequences of clade 6B.1A.5a.2a., and A197T was present in 7 sequences from this same clade. The N130K, K156N, I161L, and A250V mutations were present in all sequences of clades 6B.1A.5a.1 and 6B.1A.5b. In the NA-encoding gene, S200N, V453M and K469N were present in all sequences from clades C.5.3 and C.5.3.3. I223V, R257K, and S70N were present in only six sequences from clade C.5.3.3. T16A was present in all sequences of clade B.3.1 and L127F was present in seven sequences of this same clade.

**Tableau 1:** List of mutation HA/NA for H3N2

| **H3N2** | | | | |
| --- | --- | --- | --- | --- |
| **Mutation** | **Gene** | **Clade** | **Year** | **Number of sequences** |
| R299K | HA | 3C.2a1b.1a | 2019/2020 | 5 |
| R261Q | HA | 3C.2a1b.1 | 2019 | 9 |
| T65K | HA | 3C.2a1b.2a.2a.3a.1 | 2024 | 11 |
| N122D | HA | 3C.2a1b.2a.2a.3a.1 | 2024 | 11 |
| K276E | HA | 3C.2a1b.2a.2a.3a.1 | 2024 | 11 |
| S124N | HA | 3C.2a1b.2a.2a.3a.1 | 2024 | 11 |
| D186G | HA | 3C.2a1 | 2018 | All |
| N190D | HA | 3C.2a1 | 2018 | All |
| S193F | HA | 3C.2a1 | 2018 | All |
| A138S | HA | 3C.2a1b.1a | 2019/2020 | All |
| E50K | HA | 3C.2a1b.2a.2a.3a | 2022 | All |
| N96S | HA | 3C.2a1b.2a.2a.3a | 2022 | All |
| I192F | HA | 3C.2a1b.2a.2a.3a | 2022 | All |
| E50K | HA | 3C.2a1b.2a.2a.3a.1 | 2024 | All |
| N96S | HA | 3C.2a1b.2a.2a.3a.1 | 2024 | All |
| I192F | HA | 3C.2a1b.2a.2a.3a.1 | 2024 | All |
| I140K | HA | 3C.2a1b.2a.2a.3a.1 | 2024 | All |
| D186G | HA | 3C.2a1b.1 | 2019 | All |
| N190D | HA | 3C.2a1b.1 | 2019 | All |
| S193F | HA | 3C.2a1b.1 | 2019 | All |
| K344E | NA | A.2.2 | 2018/2019 | 7 |
| S44F | NA | A.2.2 | 2019/2020 | 6 |
| I65V | NA | B.4 | 2022/2024 | 7 |
| V263I | NA | A.2.2 | 2019/2020 | 23 |
| R400K | NA | B.4.2 | 2024 | All |
| R315S | NA | A.2.2 | 2018/2019/2020 | All |
| D346G | NA | A.2.2 | 2018/2019/2020 | All |
| N463D | NA | A.2.2 | 2018/2019/2020 | All |

**Tableau 2:** List of mutation HA/NA for H1N1

| **H1N1** | | | | |
| --- | --- | --- | --- | --- |
| **Mutation** | **Gene** | **Clade** | **Year** | **Number of sequences** |
| A197T | HA | 6B.1A.5a.2a | 2023 | 7 |
| K54Q | HA | 6B.1A.5a.2a | 2023 | All |
| A186T | HA | 6B.1A.5a.2a | 2023 | All |
| E224A | HA | 6B.1A.5a.2a | 2023 | All |
| R259K | HA | 6B.1A.5a.2a | 2023 | All |
| K308R | HA | 6B.1A.5a.2a | 2023 | All |
| N130K | HA | 6B.1A.5a.1 | 2021 | All |
| K156N | HA | 6B.1A.5a.1 | 2021 | All |
| I161L | HA | 6B.1A.5a.1 | 2021 | All |
| A250V | HA | 6B.1A.5a.1 | 2021 | All |
| N130K | HA | 6B.1A.5b | 2018 | All |
| K156N | HA | 6B.1A.5b | 2018 | All |
| I161L | HA | 6B.1A.5b | 2018 | All |
| A250V | HA | 6B.1A.5b | 2018 | All |
| I223V | NA | C.5.3.3 | 2023 | 6 |
| R257K | NA | C.5.3.3 | 2023 | 6 |
| S70N | NA | C.5.3.3 | 2023 | 6 |
| L127F | NA | B.3.1 | 2021 | 7 |
| S200N | NA | C.5.3 | 2023 | All |
| V453M | NA | C.5.3 | 2023 | All |
| K469N | NA | C.5.3 | 2023 | All |
| S200N | NA | C.5.3.3 | 2023 | All |
| V453M | NA | C.5.3.3 | 2023 | All |
| K469N | NA | C.5.3.3 | 2023 | All |
| T16A | NA | B.3.1 | 2021 | All |

**Figure 3.** Phylogenetic tree based on the segment 4 from the influenza A/H3N2 virus genomes and mutation (HA)

**Figure 4.** Phylogenetic tree based on the segment 4 and 6 from the influenza A/H3N2 virus genomes and mutation (NA)

**Figure 3.** Phylogenetic tree based on the segment 4 from the influenza A/H1N1 virus genomes and mutation (HA)

**N130K, K156N, I161L, A250V**

**K54Q A186TE224AR259KK308R**

**N130K, K156N, I161L, A250V**

**A197T**

**A197T**

**Figure 4.** Phylogenetic tree based on the segment 6 from the influenza A/H1N1 virus genomes and mutation (NA)
