# Supplementary material for: Epidemiological, Clinical and Viral Genomic Features of Influenza Virus Infections Among Pilgrims During the Grand Magal of Touba Over 7 Years (2018–2024)
Source: J Med Virol. 2026 Aug 24;98(8):e71120. doi: 10.1002/jmv.71120 (PMC13501545; doi:10.1002/jmv.71120)
Supplement: Supplementary file 2 [file JMV-98-e71120-s002.docx]

**Supplementary Table 1.** PCR primers and probes for the real-time reverse-transcription-PCR systems (a) and reverse-transcription-PCR systems (b) used to detect and amplify fragments of the influenza A and B virus genomes

**a.**

| **Targeted sequences** | **Primer and probe names** | **Primer and probe sequences** |
| --- | --- | --- |
| Matrix protein / Influenza A | FluA_F | GACCRATCCTGTCACCTCTGAC |
|  | FluaA_R | AGGGCATTYTGGACAAAKCGTCTA |
|  | FluA_P | FAM- TGCAGTCCTCGCTCACTGGGCACG |
| Hemagglutinin / Influenza B | FluB_F | AAATACGGTGGATTAAAYAAAAGCAA |
|  | FluB_R | CCAGCAATAGCTCCGAAGAAA |
|  | FluB_P | VIC-CACCCATATTGGGCAATTTCCTATGGC |

**b.**

Forward primers:

MBTuni-12: GTTACGCGCCAGCAAAAGCAGG

MBTuni-12 SNV: GTTACGCGCCAGCGAAAGCAGG

Reverse primer:

MBTuni-13: GTTACGCGCCAGTAGAAACAAGG
